# Clinical, pathological, imaging, and genetic characterization in a Taiwanese cohort with limb-girdle muscular dystrophy

**DOI:** 10.1186/s13023-020-01445-1

**Published:** 2020-06-23

**Authors:** Wen-Chen Liang, Yuh-Jyh Jong, Chien-Hua Wang, Chen-Hua Wang, Xia Tian, Wan-Zi Chen, Tzu-Min Kan, Narihiro Minami, Ichizo Nishino, Lee-Jun C. Wong

**Affiliations:** 1 Department of Pediatrics, Kaohsiung Medical University Hospital, Kaohsiung Medical University, Kaohsiung, Taiwan; 2 Translational Research Center of Neuromuscular Diseases, Kaohsiung Medical University Hospital, Kaohsiung Medical University, Kaohsiung, Taiwan; 3 Department of Pediatrics, School of Medicine, College of Medicine, Kaohsiung Medical University, Kaohsiung, Taiwan; 4 Department of Laboratory Medicine, Kaohsiung Medical University Hospital, Kaohsiung Medical University, Kaohsiung, Taiwan; 5 Graduate Institute of Clinical Medicine, College of Medicine, Kaohsiung Medical University, Kaohsiung, Taiwan; 6 Baylor Genetics, Houston, TX USA; 7 Department of Molecular and Human Genetics, Baylor College of Medicine, Houston, TX USA; 8 Department of Pathology, Kaohsiung Medical University Hospital, Kaohsiung Medical University, Kaohsiung, Taiwan; 9 Department of Laboratory Medicine, National Center Hospital, National Center of Neurology and Psychiatry, Tokyo, Japan; 10 Department of Neuromuscular Research, National Institute of Neuroscience, National Center of Neurology and Psychiatry, Tokyo, Japan; 11 Department of Genome Medicine Development, Medical Genome Center, National Center of Neurology and Psychiatry, Tokyo, Japan

**Keywords:** Limb-girdle muscular dystrophy, Muscle imaging, Alpha-dystroglycanopathy, Next-generation sequencing

## Abstract

**Background:**

Limb-girdle muscular dystrophy (LGMD) is a genetically heterogeneous, hereditary disease characterized by limb-girdle weakness and histologically dystrophic changes. The prevalence of each subtype of LGMD varies among different ethnic populations. This study for the first time analyzed the phenotypes and genotypes in Taiwanese patients with LGMD in a referral center for neuromuscular diseases (NMDs).

**Results:**

We enrolled 102 patients clinically suspected of having LGMD who underwent muscle biopsy with subsequent genetic analysis in the previous 10 years. On the basis of different pathological categories, we performed sequencing of target genes or panel for NMDs and then identified patients with type 1B, 1E, 2A, 2B, 2D, 2I, 2G, 2 N, and 2Q. The 1B patients with *LMNA* mutation presented with mild limb-girdle weakness but no conduction defect at the time. All 1E patients with *DES* mutation exhibited predominantly proximal weakness along with distal weakness. In our cohort, 2B and 2I were the most frequent forms of LGMD; several common or founder mutations were identified, including c.1097_1099delACA (p.Asn366del) in *DES*, homozygous c.101G > T (p.Arg34Leu) in *SGCA*, homozygous c.26_33dup (p.Glu12Argfs*20) in *TCAP*, c.545A > G (p.Tyr182Cys), and c.948delC (p.Cys317Alafs*111) in *FKRP*. Clinically, the prevalence of dilated cardiomyopathy in our patients with LGMD2I aged > 18 years was 100%, much higher than that in European cohorts. The only patient with LGMD2Q with *PLEC* mutation did not exhibit skin lesions or gastrointestinal abnormalities but had mild facial weakness. Muscle imaging of LGMD1E and 2G revealed a more uniform involvement than did other LGMD types.

**Conclusion:**

Our study revealed that detailed clinical manifestation together with muscle pathology and imaging remain critical in guiding further molecular analyses and are crucial for establishing genotype–phenotype correlations. We also determined the common mutations and prevalence for different subtypes of LGMD in our cohort, which could be useful when providing specific care and personalized therapy to patients with LGMD.

## Background

Limb-girdle muscular dystrophy (LGMD) refers to a group of genetically heterogeneous, hereditary muscle diseases characterized by clinically progressive girdle weakness and pathologically dystrophic change. LGMD results from defects in proteins located throughout a muscle fiber including the nucleus, sarcoplasm, sarcomere, sarcolemma, and extracellular matrix [1]. The LGMD spectrum has rapidly expanded in the past 10 years because of methodological advancements in genetic testing, such as next-generation sequencing (NGS). Currently, LGMD, divided into type 1 and 2 depending on autosomal dominant or recessive inheritance, comprises more than 30 subtypes. Clinical, pathological, or genetic analysis in isolation is typically insufficient for precise diagnosis; multidisciplinary evaluation in conjunction with genetic testing is required to achieve final diagnosis and essential for outcome prediction, comorbidity prevention, genetic counseling, and further precise mRNA-targeting or gene correction therapy [2].

LGMD prevalence varies greatly among different geographic areas and subtypes. This might partly result from the existence of founder mutation and consanguineous marriage in certain geographic regions and ethnic populations [3]. A meta-analysis on the epidemiology of muscular dystrophy estimated the prevalence of LGMD at approximately 1.63 per 100,000 [4]. In general, LGMD2A and 2B are the most common subtypes worldwide [5–9], except for some European areas, where LGMD2I and 2 L occur most frequently, and North Africa, where LGMD2C exhibited the highest prevalence [3, 10–12]. However, integrated data regarding LGMD epidemiology is sparse in Asian countries [13, 14].

In this retrospective study, we report the detailed phenotype and genotype of Taiwanese patients with LGMD in a referral center for neuromuscular diseases (NMDs), as confirmed by various mutation analyses, including NGS analysis.

## Methods

### Patients

In total, 102 patients with a suspected diagnosis of LGMD at Kaohsiung Medical University Hospital between 1996 and 2016 were enrolled. Muscle biopsies were performed on 84 patients for analysis. LGMD diagnosis was based on progressive proximal muscle weakness (age at onset > 2 years) with dystrophic changes in muscle pathology, defined as necrotic and regenerating processes with endomysial fibrosis. Subsequently, histochemistry and immunohistochemistry (IHC) studies and genetic analyses were performed for diagnostic purposes. Genomic DNA was extracted from whole blood using Puregene DNA Isolation Kit (Gentra, Minneapolis, MN, USA) according to the manufacturer’s instructions. All the information used in this study was extracted from the patients’ medical records. This study was approved by the Institutional Review Board of Kaohsiung Medical University Hospital (KMUHIRB-SV(II)-20,150,034).

### Histochemistry

Biopsied muscle specimens were snap-frozen in isopentane cooled in liquid nitrogen. A serial frozen section was stained by a battery of histochemical stains including hematoxylin and eosin (H&E), modified Gomori trichrome (mGt) and NADH-tetrazolium reductase (NADH-TR).

### Immunohistochemistry

Muscle biopsy cryosections of 6-μm thickness were immunostained using commercially available antibodies against dystrophin, dysferlin, alpha-, beta-, gamma, and delta-sarcoglycans (Leica, IL, USA), alpha-dystroglycan (Merk Millipore, MA, USA) according to standard protocols with a Ventana Benchmark automated stainer [15].

### Muscle computed tomography

Computed tomography (CT) was performed with a 5-mm slice width and 1-s rotation time using the Brilliance 64 apparatus (Philips Medical Systems, Haifa, Israel) operating at 120 kV and 200 mA. Slices were obtained transaxially at the shoulder joints, the mid-position of the upper arm, the hip joints, the mid-position of the thigh, and the maximum circumference of the calf. The field of view was 35 cm with a 512 × 512 matrix. For each patient, individual muscles were evaluated with a 4-point semiquantitative visual scale, originally proposed by Lamminen et al. [16], as follows: grade I, normal muscle (grade value = 1); grade II, slightly (< 50%) hypointense, patchy intramuscular signal change (grade value = 2); grade III, markedly hypointense (> 50%), patchy, intramuscular signal change (grade value = 3); and grade IV, homogeneous hypointense signal in the whole muscle (grade value = 4). Changes were considered significant if present in more than two slices.

### NGS analysis

The capture probe library contains 247 genes related to a broad spectrum of NMDs [17, 18], such as congenital myopathy, congenital muscular dystrophy, congenital myasthenic syndrome, motor neuron disease, arthrogryposis multiplex congenita, and other myopathies. Of them, 44 genes (Supplement Table 1) are known to be responsible for LGMD. All coding exons and at least 20 bp of the flanking intronic sequences of target genes were captured and sequenced on Illumina HiSeq2000. The sequence alignment, analytical pipeline, and variant calling have been published previously [17, 18].

## Results

**Fig. 1:**
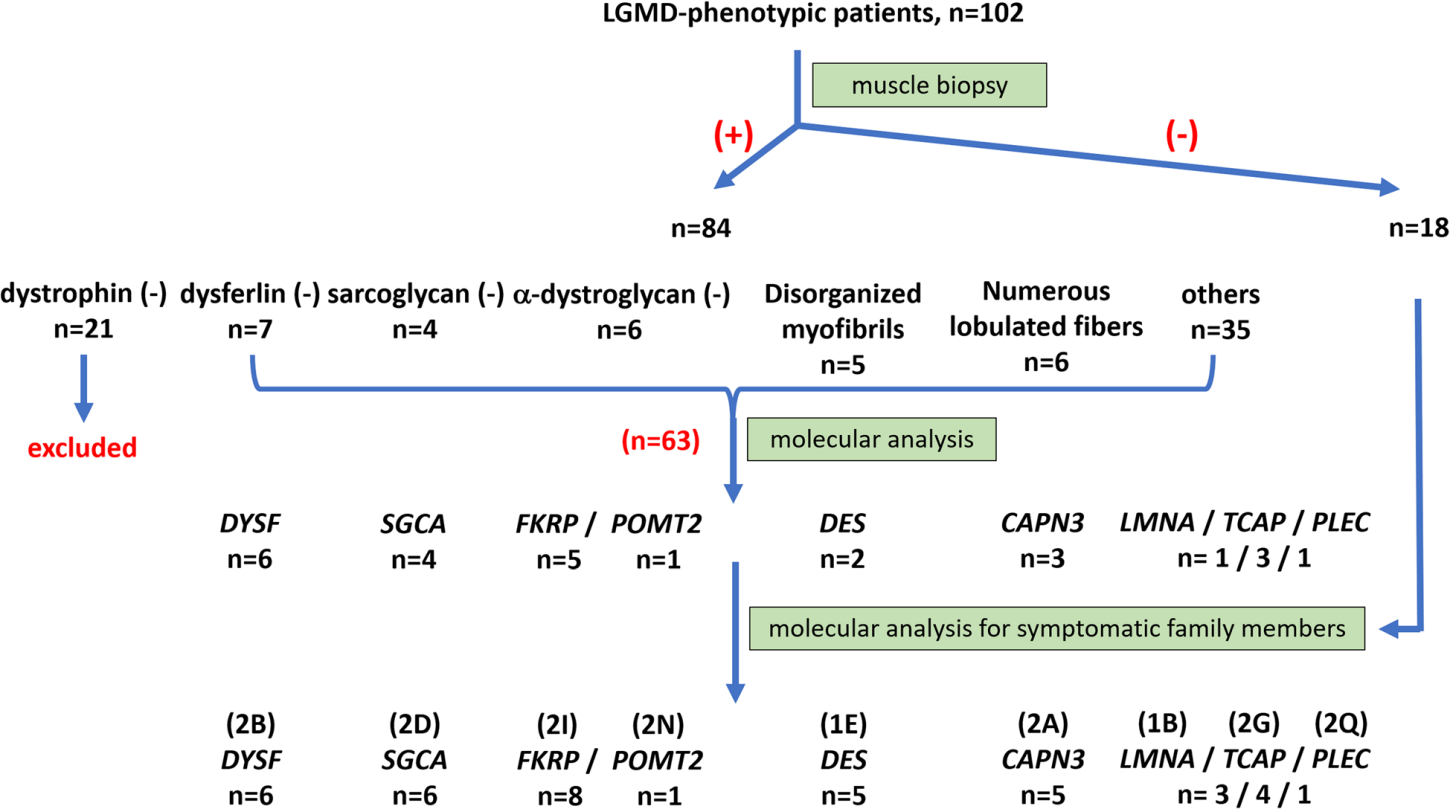
Flow of diagnosis process

On the basis of muscle pathology, patients were first categorized into seven groups according to dystrophic changes with (1) dystrophin deficiency, (2) dysferlin deficiency, (3) sarcoglycan deficiency, (4) alpha-dystroglycan deficiency, (5) markedly disorganized myofibrillar network with or without cytoplasmic body and rimmed vacuole, (6) numerous lobulated fibers, and (7) others (Fig. 1). For group 1 (*n* = 21), following genetic analysis of DMD have confirmed the diagnosis of dystrophinopathy for these patients, including 13 Duchenne-type and 8 Becker-type muscular dystrophy. Group 7 was defined as dystrophic change without the characters of group 1 to 6. Excluding group 1, subsequent direct sequencing for target genes or NGS panel for LGMD were performed on the remaining patients with (*n* = 63) and without (*n* = 18) muscle pathology. Pathogenic/likely pathogenic mutations and variants of unknown significance were identified in 39 patients [19]; the clinical, pathological, imaging, and molecular results are summarized in Table 1.

**Table 1:** Summary of patients with LGMD

Subtype (*gene*)	Patient No.	Age (Y)/Gender	Onset age (Y)	Initial weakness	calf hyper−/atrophy	Loss of ambu-lation (Y)	Cardiac/pulmonary involvement	CK level (IU/L) (age at first visit)	Muscle pathology (category No)	Mutation (Allele 1)	Mutation (Allele 2)
1B (*LMNA*)	P1	5/M^	Uncertain^%^	P	No/No	Not yet	No/No	1632 (2)	7	c.1357C > T (p.Arg453Trp) *LP*	
	P2	7/F^	Uncertain^%^	P	No/No	Not yet	No/No	2718 (5)	ND	c.1357C > T (p.Arg453Trp) *LP*	
	P3	30/F^	Uncertain	P	No/No	Not yet	unknown	ND	ND	c.1357C > T (p.Arg453Trp) *LP*	
1E (*DES*)	P4	59/F^	50	P + D	No/No	Not yet	No/No	ND	ND	c.1097_1099delACA (p.Asn366del) *P*	
	P5	45/F^	35	P + D	No/No	Not yet	No/No	474 (35)	ND	c.1097_1099delACA (p.Asn366del) *P*	
	P6	42/M^	28	P - > D	No/No	Yes (40)	Yes/Yes	1458 (32)	5	c.1097_1099delACA (p.Asn366del) *P*	
	P7	55/F^#^	43	P + D	No/No	Not yet	No/Yes	93 (51)	5	c.1097_1099delACA (p.Asn366del) *P*	
	P8	28/F^#^	28	P	No/No	Not yet	No/No	591 (26)	ND	c.1097_1099delACA (p.Asn366del) *P*	
2A (*CAPN3*)	P9	41/F	15	P	No/Yes	Yes (36)	No/Yes	914 (33)	6	c.2047_2050del4 (p.Lys683fs) *LP*	c.2047_2050del4 (p.Lys683fs) *LP*
	P10	42/F^	13	P	No/No	Yes (35)	No/No	432 (35)	6	c.1621C > T (p.Arg541Trp) *P*	c.2305C > T (p.Arg769Trp) *LP*
	P11	40/F^	14	P	Unknown	Yes (29)	Unknown	ND	ND	c.1621C > T (p.Arg541Trp) *P*	c.2305C > T (p.Arg769Trp) *LP*
	P12	24/M	10	P	No/Yes	Yes (23)	No/Yes	2004 (20)	6	c.1837A > T (p.Lys613*) *VUS*	c.2050 + 1G > A *P*
	P13	53/F	30	P	No/Yes	Yes (49)	No/No	361 (49)	ND	c.1016C > G (p.Thr339Arg) *VUS*	c.1817C > T (p.Ser606Leu) *LP*
2B (*DYSF*)	P14	22/M	14	P	No/No	Not yet	No/No	14,509 (13)	2	c.2311C > T (p.Gln771*) *P*	c.2870_2874delAGACC (p.Gln957Profs*12) *P*
	P15	41/F	20	P> > D	No/Yes	Yes (36)	No/No	5025 (34)	2	exon 5 del *P*	exon 5 del *P*
	P16	52/F	15	P> > D	No/Yes	Yes (48)	No/Yes	4250 (26)	2	no mutation identified	no mutation identified
	P17	41/M	15	P + D	No/Yes	Yes (uncertain)	No/No	16,020 (21)	2	c.2870_2874delAGACC (p.Gln957Profs*12) *P*	c.5302C > T (p.Arg1768Trp) *P*
	P18	25/M	13	P + D	No/Yes	Not yet	No/No	11,972 (13)	2	c.937 + 1G > A *P*	c.1721 T > C (p.Leu574Pro) *LP*
	P19	54/M	18	P	unknown	Yes (42)	No/No	707 (48)	ND	c.937 + 1G > A *P*	c.937 + 1G > A *P*
	P20	18/F	16	P	No/No	Not yet	No/No	5403 (17)	2	c.965 T > C (p.Leu322Pro) *LP*	c.2311C > T (p.Gln771*) *P*
2D (*SGCA*)	P21	23/M	3	P	Yes/No	Yes (10)	Yes/Yes	39,072	3	c.101G > T (p.Arg34Leu) *P*	c.101G > T (p.Arg34Leu) *P*
	P22	25/F	5	P	Yes/No	Yes (15)	Yes/Yes	21,332	3	c.101G > T (p.Arg34Leu) *P*	c.101G > T (p.Arg34Leu) *P*
	P23	49*/M	< 5	P	unknown	Yes (28)	Unknown/Yes	unknown	ND	c.101G > T (p.Arg34Leu) *P*	c.101G > T (p.Arg34Leu) *P*
	P24	14/M	6	P	Yes/No	Not yet	No/No	11,042	3	c.101G > T (p.Arg34Leu) *P*	c.101G > T (p.Arg34Leu) *P*
	P25	6/M	Not yet	P	Yes/No	Not yet	No/No	20,400	ND	c.101G > T (p.Arg34Leu) *P*	c.101G > T (p.Arg34Leu) *P*
	P26	3/M	Not yet	P	Yes/No	Not yet	No/No	9800	3	c.101G > T (p.Arg34Leu) *P*	c.101G > T (p.Arg34Leu) *P*
2G (*TCAP*)	P27	61/F	12	P	No/No	Yes (42)	No/Yes	474 (55)	7	c.26_33dup (p.Glu12Argfs*20) *P*	c.26_33dup (p.Glu12Argfs*20) *P*
	P28	59/M	15	P	Unknown	Yes (33)	Yes/Yes	ND	ND	c.26_33dup (p.Glu12Argfs*20) *P*	c.26_33dup (p.Glu12Argfs*20) *P*
	P29	35/M	21	P	Yes/No	Yes (33)	No/No	1441 (24)	7	c.26_33dup (p.Glu12Argfs*20) *P*	c.26_33dup (p.Glu12Argfs*20) *P*
	P30	36/F	30	P + D	Yes/No	Not yet	No/No	1403 (33)	7	c.26_33dup (p.Glu12Argfs*20) *P*	c.26_33dup (p.Glu12Argfs*20) *P*
2I (*FKRP*)	P31	42/F	2	P	Yes/No	Yes (38)	Yes/Yes	1–1.5 K (30)	4	c.263A > T (p.Tyr88Phe) *LP*	c.263A > T (p.Tyr88Phe) *LP*
	P32	23/M	5	P	Yes/No	Yes (20)	Yes/Yes	> 10 K (12)	4	c.545A > G (p.Tyr182Cys) *P*	c.948delC (p.Cys317Alafs*111) *P*
	P33	47*/M^	10	P	Yes/No	Yes (29)	Yes/Yes	1.5-2 K (31)	4	c.545A > G (p.Tyr182Cys) *P*	c.948delC (p.Cys317Alafs*111) *P*
	P34	38/M^	17	P	Yes/No	Yes (33)	Yes/Yes	1.5-2 K (30)	ND	c.545A > G (p.Tyr182Cys) *P*	c.948delC (p.Cys317Alafs*111) *P*
	P35	18/M	2	P	Yes/No	Yes (15)	No/No	6-9 K (10)	4	c.823C > T (p.Arg275Cys) *P*	c.948delC (p.Cys317Alafs*111) *P*
	P36	31/F	2	P	Yes/No	Yes (12)	Yes/Yes	> 10 K (3)	4	c.823C > T (p.Arg275Cys) *P*	c.948delC (p.Cys317Alafs*111) *P*
	P37	42/F^#^	10	P	Yes/No	Yes (38)	Yes/Yes	3 K (38)	4	c.545A > G (p.Tyr182Cys) *P*	c.151G > T (p.Val51Phe) *VUS*
	P38	25*/M^#^	12	P	Yes/No	No	Yes/Yes	unknown	ND	c.545A > G (p.Tyr182Cys) *P*	c.151G > T (p.Val51Phe) *VUS*
2 N (*POMT2*)	P39	M/41		P	Yes/No	Yes (35)	No/Yes	1862 (33)	4	c.1061A > G (p.Tyr354Cys) *VUS*	c.1061A > G (p.Tyr354Cys) *VUS*
2Q (*PLEC*)	P40	M/29	3	P	No/No	Not yet	No/Yes	513 (6)	7	c.12731G > A (p.Arg4244His) *VUS*	c.7928A > G (p.Glu2643Gly) *VUS*

* expired; ^ and ^#^ siblings or family members; ^%^ in foster family; *D* distal, *F* female; *K* ×10^3^, *M* male, *ND* not done, *P* proximal, *Y* year, *P* pathogenic, *LP* likely pathogenic, *VUS* variant of unknown significance (Ref [18])

### LGMD1

In pathology group 7, a reported mutation, Arg453Trp [20], in *LMNA* (LGMD1B) was identified in an index patient whose mother and elder sister were also symptomatic patients. All had onset in early childhood, mild proximal weakness with almost nonprogressive course and were still independently ambulant. No significant cardiac involvement has been identified thus far.

In pathology group 5, we identified two unrelated index patients with *DES* mutation (LGMD1E). When expanding the mutation analysis to symptomatic family members, we further identified the same mutation in four and six patients in two different families. Of a total of 12 patients, 5 exhibited initially proximal-predominance or mixed proximal and distal weakness, categorized as LGMD1E, and 7 presented with initially distal-predominance weakness, compatible with typical myofibrillar myopathy. Clinical information is summarized in Table 1.

### LGMD2

In pathology group 6, we identified *CAPN3* mutations (LGMD2A) in five patients from four families with onset in the early teens and age at loss of walking ability in the third to fourth decades of life except for one patient who had later onset and slower progression and had been misdiagnosed as having polymyositis and been treated with steroids for several years. In all patients, diffuse muscle atrophy was prominent, particularly in the gluteal, posterior thigh and calf muscles. Numerous lobulated fibers are hallmark pathological features in LGMD2A [21].

Complete dysferlin deficiency (pathological group 2), revealed by IHC, leads to the diagnosis of LGMD2B. *DYSF* mutations were later identified in six patients. No mutation was identified in one patient categorized as group 2. The age at onset was in the late teens or young adulthood and the age of losing walking ability was approximately 30 years after disease onset. Creatine kinase (CK) was typically more than 10,000 IU/L even during the asymptomatic stage. Muscle pathology in our patients did not reveal overt inflammatory cell infiltration as some previous literature reported [22].

In pathology group 3, we previously reported five patients from a five-generation family carrying a homozygous founder mutation, c.101G > T/p.Arg34Leu in *SGCA* (LGMD2D) [23]. We later diagnosed the sixth patient from another aboriginal family who harbored the same homozygous mutation, again suggesting the probable high carrier frequency of this mutation in the aboriginal population in Taiwan.

In pathology group 7, three patients from two families with a homozygous *TCAP* mutation (LGMD2G) have been reported previously [17]; one additional patient with the same mutation was later diagnosed. The disease onset and progression were similar to those of LGMD2B, but asymmetric involvement was recorded. No prominent scoliosis was noted even in the advanced stage but dropped foot was observed in the ambulatory phase. Muscle pathology revealed mild dystrophic and vacuolar change. All patients carried the same mutation, c.26_33dup(p.Glu12Argfs*20), homozygously.

In pathology group 4, six patients with *FKRP* mutations (LGMD2I) from five families have been reported [15]. We diagnosed an additional two sibling patients subsequently. c.545A > T, a common mutation in our cohort, has also been reported as a common mutation in other studies [24, 25]. Moreover, one patient with LGMD2 N with *POMT2* mutations was identified, presenting with early childhood onset and slowly progressive weakness with asymmetric involvement. He exhibited calf, triceps hypertrophy and winged scapula, and his younger sister had similar symptoms but could not be approached.

In group 7, one patient with LGMD2Q with PLEC mutations was identified. He presented with onset in early childhood and slow progression. He was still ambulant with neck, mild proximal muscle, and facial weakness. He did not reveal any skin lesions or gastrointestinal problems.

### Cardiopulmonary function

In contrast to LGMD1, no significant cardiopulmonary dysfunction was observed in LGMD2A, 2B, and 2G even after losing ambulation. Longer follow-up duration may be necessary to further identify potential cardiac and respiratory problems. Notably, all patients with LGMD2I, except for the youngest one (currently aged 17 years), have developed dilated cardiomyopathy and significant respiratory insufficiency even when some of them remain ambulant; the prevalence of cardiac involvement in our cohort was higher than that in European reports [26, 27], although the deterioration rate of ejection fraction was similar [28]. One of our patients with LGMD2I had a cerebral infarction at the age of 20 years but the exact cause remained unclear. The patient with LGMD2 N did not develop cardiomyopathy but exhibited a considerable decrease in forced vital capacity at the age of 33 years (first visit). Our hospital has no follow-up data for this patient; therefore, no updated information is available regarding his cardiac involvement. The only patient with LGMD2Q did not exhibit cardiac involvement (at the time) but mild to moderate restrictive pulmonary defect had been observed previously.

### Muscle imaging

**Fig. 2:**
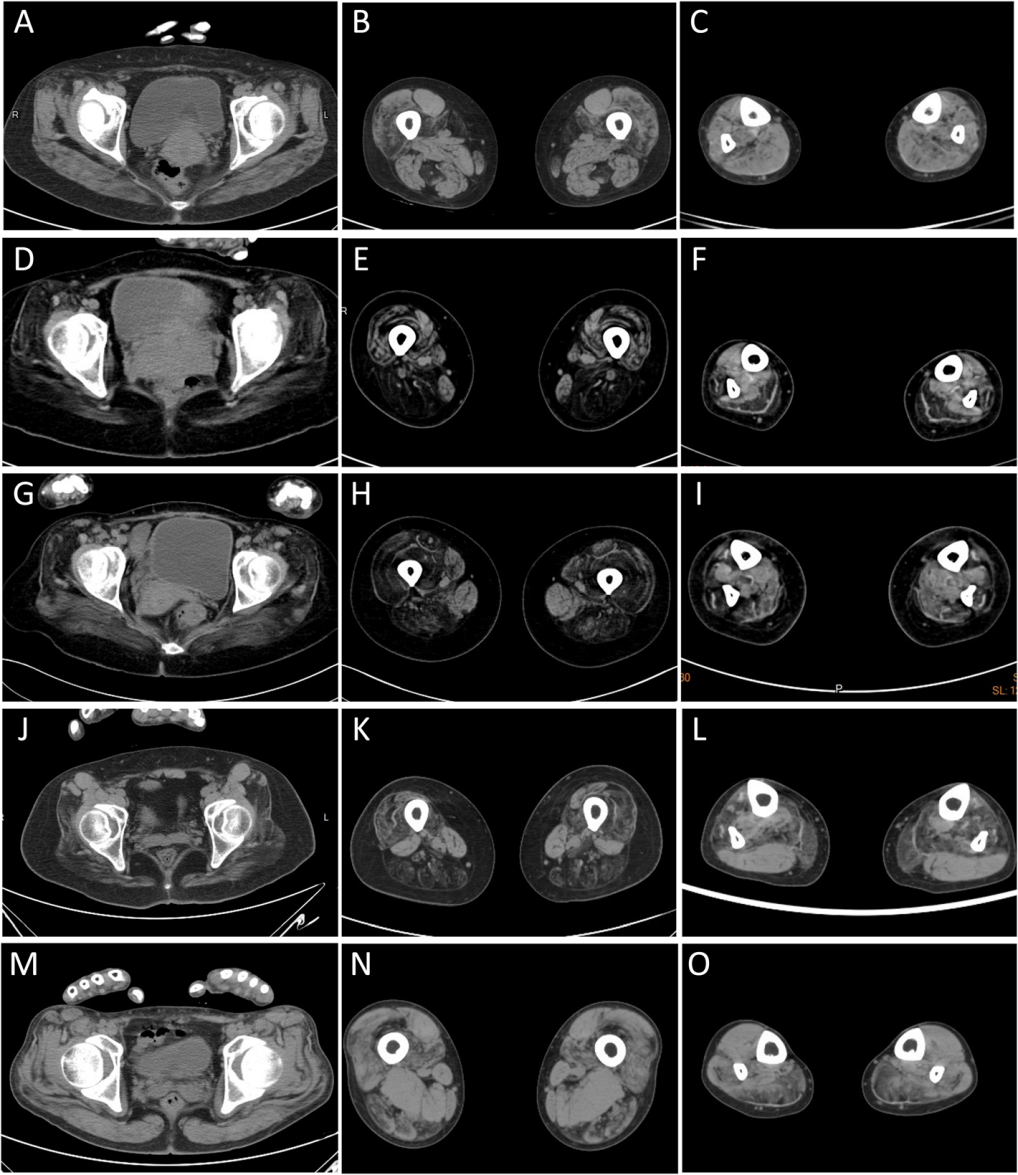
Muscle imaging of LGMD1E, 2A, 2B, 2G, and 2Q. Muscle CT images of 1E revealed most involvement in the S and ST, and less involvement in the VL and VIM at thigh level and the SO, TA, and TP muscles at calf level (**a**, **b**, **c**). Compared with LGMD2A (**d**, **e**, **f**), gluteal muscles were less affected but the TA was more severely involved in LGMD2B (**g**, **h**, **i**). LGMD2G exhibited severe involvement of the gluteus, long head of the BF, AM, ST, SM, SO, medial head of GC, and TA muscles; the short head of BF, AL, S, FD, HL, and lateral head of GC muscles were relatively spared (**j**, **k**, **l**). For LGMD2Q (**m**, **n**, **o**), in the thigh, the AM as well as S and G were relatively hypertrophic and preserved; by contrast, the ST, SM, and QF (except for RF) were markedly affected. At calf level, the GC and P were involved, whereas the TA and EL muscles were preserved and slightly hypertrophic. (AM: adductor magnus; AL: adductor longus; BF: biceps femoris; EL: extensor longus; FD: flexor digitorum; G: gracilis; GC: gastrocnemius; HL: hallucis longus; P: peroneus; QF: quadriceps femoris; RF: rectus femoris; S: sartorius; SO: soleus; ST: semitendinosus; SM: semimembranosus; TA: tibialis anterior; TP: tibialis posterior; VL: vastus lateralis; VIM: vastus intermedius)

The muscle involvement in LGMD1E (Fig. 2a, b, and c) was relatively characteristic in the early stage, revealing semitendinosus involvement solely, then progressing to the sartorius and vastus intermedius in the thigh; in the lower legs, calf involvement was generally mild but most in the tibialis anterior. Compared with the findings in LGMD2A (Fig. 2d, e, and f), LGMD2B (Fig. 2g, h, and i) revealed less involvement in gluteal muscles but more in the lower limbs, including the soleus, gastrocnemius (most severely affected with mean grade value 4), and tibialis, which seemed to be affected first; however, no foot drop was observed concurrently. The aforementioned findings suggest an overlap between LGMD2B and Miyoshi myopathy. Three patients with LGMD2G underwent muscle imaging examinations, which revealed that the adductor magus, semitendinosus, semimembranosus, and soleus muscles were affected earlier than the gluteal and tibialis anterior muscles. The shoulder girdle, short head of biceps femoris, sartorius, flexor digitorum/hallucis longus, and lateral head of the gastrocnemius muscles were relatively spared (Fig. 2j, k, and l). The muscle CT findings of patients with LGMD2I were described in our previous report [15]. However, some differences were observed between patients with LGMD2N and patients with LGMD2I. In the patients with LGMD2N, the posterior compartment of the thigh was near homogeneously affected including the gracilis, whereas it was relatively preserved in patients with LGMD2I. Muscle involvement in the only patient with LGMD2Q was peculiar (Fig. 2m, n, and o). In the thigh, the adductors as well as the rectus femoris, sartorius, and gracilis were relatively hypertrophic and preserved; by contrast, the long head of the biceps femoris, semitendinosus, and semimembranosus were markedly affected. At the lower leg level, the gastrocnemius and peroneus were involved, whereas the anterior tibialis and extensor longus muscles were preserved and were slightly hypertrophic. The major significant findings were summarized in Table 2.

**Table 2:** Muscle imaging findings: muscle involvement in lower extremities

	Thigh	Lower leg
LGMD1E	ST = > S, VM, VIM, VL, GM	TA
LGMD2A	SM, ST, BF, AM, GM = > VL, VIM	GC = > EL, P
LGMD2B	SM, ST, BF, AM = > GM, VL, VIM	GC = > P, TA
LGMD2D	SM, ST, BF, AM, GM = > VL	GC = > SO, EL
LGMD2G	AM, ST, SM = > GM	SO = > TA
LGMD2I	GM = > BF, AM, AL = > QF	GC, SO

=> progression direction, *AM* adductor magnus, *AL* adductor longus, *BF* biceps femoris, *EL* extensor longus, *FD* flexor digitorum, *G* gracilis, *GC* gastrocnemius, *GM* gluteus maximus, *P* peroneus, *QF* quadriceps femoris, *S* sartorius, *SO* soleus, *ST* semitendinosus, *SM* semimembranosus, *TA* tibialis anterior, *VL* vastus lateralis, *VIM* vastus intermedius, *VM* vastus medialis

## Discussion

NGS is widely used for molecular diagnosis of NMDs. The target gene panel approach remains the standard approach, but the utility of whole exome sequencing is increasingly common particularly in patients with atypical phenotype or negative panel analysis results. However, with only NGS results, conclusively confirming the pathogenic variant is difficult. Detailed clinical, biochemical, electrophysiological, and pathological investigations remain critical to establish genotype-phenotype correlation for accurate final diagnosis [18, 29]. Taking patient 5 as an example, the patient was diagnosed as LGMD2B first based on completely deficient dysferlin expression on IHC; however the following NGS analysis identified two unreported compound heterozygous variants in *SGCA* but not in *DYSF*. We thus performed IHC, western blot and target sequencing again for both genes, and finally confirmed that homozygous exon 5 deletion of *DYSF* was the cause of disease as all sarcoglycans expression showed normal. This case does emphasize that the consistency between DNA defect and protein expression is important to judge the causative gene of LGMD.

In our cohort, all four patients with LGMD2G carried the same homozygous hot spot mutation, c.26_33dup (p.Glu12Argfs*20), which has previously been reported only in three Chinese patients and one Indian patient [30, 31]. Taken together, our results further emphasize the potential founder effect of this mutation in East and South Asia. A recent study focusing on this *TCAP* mutation demonstrated promising therapeutic results by gene editing using patient-derived induced pluripotent stem cells, highlighting the importance of accurate molecular diagnosis for patients with hereditary muscle diseases [2].

Clinically, LGMD2A might appear similar to LGMD2B in some aspects, but patients with LGMD2B exhibit a milder disease course compared with patients with LGMD2A; however, they often have higher CK levels of more than 10,000 IU/L. Gluteal muscle involvement is relatively low in LGMD2B; therefore, hip extension ability could be a distinguishing characteristic between these two types in physical examinations. Furthermore, cardiopulmonary dysfunction has been reported more frequent than previous reports in LGMD2A and 2B in Japan [32, 33]; however, no significant cardiopulmonary involvement was observed in our cohort. In addition, the LGMD2A patient with exceptionally mild symptoms had been taking steroids for a long period. Steroid use could ameliorate or delay disease progression, although no related reports exist. Additional studies could be considered to elucidate its potential benefit.

To date, only a few patients with LGMD2N and 2Q have been reported. According to previous literature, patients with LGMD2N exhibit a wide variety of clinical severity. Muscle magnetic resonance imaging (MRI) with cognitive function testing was proposed to differentiate LGMD2N from LGMD2A [34]. A similar approach could be used to differentiate between LGMD2N and other types of alpha-dystroglycanopathy with LGMD phenotype as some differences were noted between LGMD2I and 2N in our cohort. However, more patients with LGMD2N should be enrolled for analysis. The patient with LGMD2Q did not exhibit any skin lesions and facial involvement was only reported in one family [35].

In this cohort, rather than MRI, we used CT as the main tool for evaluating muscle disease because its operating cost and waiting time are considerably lower. In the upper extremities, the scapularis, biceps/triceps, infraspinatus/trapezium, and supraspinatus/infraspinatus were most involved in LGMD2A, 2B, 2D and 2I, respectively. In the one patient with LGMD2N, the supraspinatus, infraspinatus, rhomboid, and trapezium were mainly affected, similar to the pattern in 2I patients. Except for type 2B, muscles related to shoulder rotation (the supraspinatus and subscapularis) were more affected. The deltoid and rhomboid muscles were relatively spared in all the subtypes of LGMD2 analyzed here. This phenomenon contrasted with the image results in facioscapulohumeral muscular dystrophy, which revealed most involvement in the deltoid muscle [36]. We compared LGMD2A and 2B in terms of involvement in the lower extremities and observed more involvement of the gastrocnemius and hip adductor, but less involvement of the gluteus maximum in patients with LGMD2B. The imaging results in patients with LGMD2G have revealed similar involvement patterns among all patients, suggesting that muscle imaging could be helpful to support the diagnosis of LGMD2G. In addition, muscle imaging in our study revealed a peculiar muscle involvement pattern in patients with LGMD2Q, which has not yet been well clarified. Specific muscle involvement information enhances the differential diagnostic value of muscle imaging for LGMD patient.

This study comprehensively analyzed Taiwanese patients clinically suspected to have LGMD and provided significant information for making final diagnosis. From the results, the most common subtypes of LGMD in our cohort are 2A, 2B and 2I which is similar to the reports from other countries. However, as the mutations identified in 2D and 2G are most likely founder mutations, the incidence of these two subtypes of LGMD may be higher than other regions but further studies would be helpful to confirm this hypothesis. Furthermore, as the retrospective time is as long as 20 years, the diagnostic approach especially about molecular analysis is different. We performed direct target gene sequencing for those with complete protein deficiency such as dysferlin and sarcoglycans in the first years, therefore, it remains a possibility that we still overlook the real mutations although we did have established strong genotype-phenotype correlation for these patients. Hence for the remaining undiagnosed LGMD patients in this study, further assays including whole exome, whole genome or transcriptome sequencing would be necessary.

## Conclusion

We described the clinical, pathological, imaging, and genetic features of patients with LGMD in Taiwan for the first time. Although NGS has been widely used for diagnosing NMDs, some limitations hinder the identification of all genetic defects in all patients. Therefore, the clinical, pathological, and imaging characteristics identified in this study may facilitate differential diagnoses, establishment of phenotype–genotype correlations and potentially personalized therapy in the future.

## Supplementary information

**Additional file 1.**

## Data Availability

The datasets used in this study are available from the corresponding author upon request.

## Abbreviations

LGMD: Limb-girdle muscular dystrophy
H&E: Hematoxylin and eosin
mGt: Modified Gomori trichrome
NADH-TR: NADH-tetrazolium reductase
IHC: Immunohistochemistry
CT: Computed tomography
NGS: Next-generation sequencing
NMD: Neuromuscular disease
