# Supplementary material for: Clinical, pathological, imaging, and genetic characterization in a Taiwanese cohort with limb-girdle muscular dystrophy
Source: Orphanet J Rare Dis. 2020 Jun 23;15:160. doi: 10.1186/s13023-020-01445-1 (PMC7310488; doi:10.1186/s13023-020-01445-1)
Supplement: Supplementary file 1 — Additional file 1. [file 13023_2020_1445_MOESM1_ESM.docx]

Supplement Table 1

| Name of panel | No. | *Genes* | | | | | | |
| --- | --- | --- | --- | --- | --- | --- | --- | --- |
| Metabolic Myopathies Panel | 55 genes | *ABHD5* | *ACAD9* | *ACADM* | *ACADS* | *ACADVL* | *AGL* | *AGPAT2* |
|  |  | *ALDOA* | *BTD* | *BSCL2* | *CAV1* | *CPT1A* | *CPT1B* | *CPT2* |
|  |  | *DLD* | *ENO3* | *ETFA* | *ETFB* | *ETFDH* | *FLAD1* | *G6PC* |
|  |  | *GAA* | *GBE1* | *GYG1* | *GYS1* | *HADH* | *HADHA* | *HADHB* |
|  |  | *LDHA* | *LPIN1* | *PDHA1* | *PDHB* | *PDHX* | *PDP1* | *PFKM* |
|  |  | *PGAM2* | *PGK1* | *PGM1* | *PHKA1* | *PHKA2* | *PHKB* | *PHKG2* |
|  |  | *PNPLA2* | *PNPLA8* | *PRKAG2* | *PTRF* | *PYGM* | *PYGL* | *RBCK1* |
|  |  | *SLC22A5* | *SLC25A20* | *SLC25A32* | *SLC37A4* | *TAZ* | *TYMP* |  |
| Congenital Muscular Dystrophy Panel | 31 genes | *ACTA1* | *ALG13* | *B3GALNT2* | *B3GNT1* | *CACNA1S* | *CHKB* | *COL6A1* |
|  |  | *COL6A2* | *COL6A3* | *DNM2* | *DPM1* | *DPM2* | *FHL1* | *FKRP* |
|  |  | *FKTN* | *GMPPB* | *POMGNT2* | *ISPD* | *ITGA7* | *LAMA2* | *LARGE* |
|  |  | *LMNA* | *POMK* | *POMT1* | *POMT2* | *POMGNT1* | *SEPN1* | *TCAP* |
|  |  | *TMEM5* | *TRAPPC11* | *TRIP4* |  |  |  |  |
| Congenital Myopathy Panel | 30 genes (4) | *ACTA1* | *BIN1* | *CACNA1S* | *CCDC78* | *CFL2* | *CNTN1* | *DNM2* |
|  |  | *HACD1* | *HNRNPA1* | *KBTBD13* | *KLHL40* | *KLHL41* | *LMOD3* | *MEGF10* |
|  |  | *MTM1* | *MTMR14* | *MYBPC3* | *MYF6* | *MYH2* | *MYH7* | *NEB* |
|  |  | *RYR1* | *SCN4A* | *SEPN1* | *SPEG* | *TNNT1* | *TPM2* | *TRIM32* |
|  |  | *TTN* | *TPM3* |  |  |  |  |  |
| Other Myopathies Panel | 48 genes (8) | *ACVR1* | *ADCK3* | *ADSSL1* | *AMPD1* | *AMPD3* | *ANO5* | *BAG3* |
|  |  | *CAV3* | *CLN3* | *COQ2* | *COQ6* | *COQ9* | *CRYAB* | *DES* |
|  |  | *DNM2* | *DYSF* | *FHL1* | *FLNC* | *GATM* | *GDF8* | *GNE* |
|  |  | *KLHL9* | *ISCU* | *KY* | *LAMP2* | *LDB3* | *MATR3* | *MEGF10* |
|  |  | *MYH7* | *NEB* | *PABPN1* | *PDSS1* | *PDSS2* | *PLEC* | *PYROXD1* |
|  |  | *RYR1* | *RYR2* | *SEPN1* | *SGCE* | *STAC3* | *STIM1* | *TIA1* |
|  |  | *TRIM54* | *TRIM63* | *MYOT* | *TTN* | *VCP* | *VMA21* |  |
| Congenital Myasthenic Syndrome Panel | 28 genes (3) | *AGRN* | *AK9* | *ALG14* | *ALG2* | *CHAT* | *CHRNA1* | *CHRNB1* |
|  |  | *CHRND* | *CHRNE* | *CHRNG* | *COL13A1* | *COLQ* | *DOK7* | *DPAGT1* |
|  |  | *GFPT1* | *GMPPB* | *LAMB2* | *LRP4* | *MUSK* | *MYO9A* | *PLEC* |
|  |  | *PREPL* | *RAPSN* | *SCN4A* | *SLC5A7* | *SLC18A3* | *SNAP25* | *SYT2* |
| Myotonic Syndromes & Ion Channel Diseases Panel | 22 genes (3) | *ATP2A1* | *CACNA1A* | *CACNA1S* | *CAV3* | *CLCN1* | *DMPK* | *HSPG2* |
|  |  | *KCNA1* | *KCNE1* | *KCNE2* | *KCNE3* | *KCNH2* | *KCNJ2* | *KCNJ5* |
|  |  | *KCNJ11* | *KCNJ18* | *KCNQ1* | *LIFR* | *SCN4A* | *SCN4B* | *SCN5A* |
|  |  | *ZNF9=CNBP* |  |  |  |  |  |  |
| Muscular Dystrophy Panel | 44 genes (22) | *ANO5* | *BVES* | *CAPN3* | *CAV3* | *DAG1* | *DES* | *DMD* |
|  |  | *DNAJB6* | *DPM3* | *DUX4* | *DYSF* | *EMD* | *FHL1* | *FKRP* |
|  |  | *FKTN* | *GAA* | *GMPPB* | *HNRNPDL* | *ISPD* | *LIMS2* | *LMNA* |
|  |  | *MYH7* | *MYOT* | *PLEC* | *POGLUT1* | *POMGNT1* | *POMT1* | *POMT2* |
|  |  | *PTRF* | *SGCA* | *SGCB* | *SGCD* | *SGCG* | *SMCHD1* | *SYNE1* |
|  |  | *SYNE2* | *TCAP* | *TMEM43* | *TNPO3* | *TOR1AIP1* | *TRAPPC11* | *TRIM32* |
|  |  | *TTN* | *VCP* |  |  |  |  |  |
| Motor Neuron Disease Panel | 30 genes | *AARS* | *ALS2* | *ANG* | *AR* | *ASAH1* | *ASCC1* | *ATP7A* |
|  |  | *ATXN2* | *BICD2* | *BSCL2* | *C9ORF72* | *CHCHD10* | *CHMP2B* | *DCTN1* |
|  |  | *DNAJB2* | *DYNC1H1* | *ELP3* | *ERBB3* | *EXOSC3* | *EXOSC8* | *FBXO38* |
|  |  | *FIG4* | *FUS* | *GARS* | *GLE1* | *HEXB* | *HNRNPA1* | *HNRNPA2B1* |
|  |  | *HSPB1* | *SMN1* |  |  |  |  |  |

The number in brackets indicates “the number of overlapping genes with other panel”; the total number of genes is 247 deducting the overlapping ones.
